# Effectiveness of Cardiovascular Evaluations and Interventions on Fall Risk: A Scoping Review

**DOI:** 10.1007/s12603-019-1165-2

**Published:** 2019-02-07

**Authors:** S. Luiting, S. Jansen, L.J. Seppälä, J.G. Daams, Nathalie van der Velde

**Affiliations:** 1 Department of Internal Medicine, Section of Geriatric Medicine, Amsterdam University Medical Center, University of Amsterdam, Amsterdam, the Netherlands; 2 Medical library, Amsterdam University Medical Center, Amsterdam, the Netherlands; 3 Department of Internal Medicine, Section of Geriatric Medicine, Amsterdam University Medical Center, Meibergdreef 9, 1105AZ, Amsterdam, the Netherlands

**Keywords:** Aged, falls, cardiovascular, interventions, evaluations

## Abstract

**Objective:**

cardiovascular abnormalities are consistently associated with fall risk in older people. However, little research has been done to assess the effect of cardiovascular interventions on fall risk. The aim of this scoping review is to explore the current literature on the effectiveness of cardiovascular evaluations and interventions in reducing fall risk in older people.

**Design:**

scoping review.

**Data sources:**

Medline, Cochrane Library, and WHO ICTRP Search Portal were systematically searched.

**Selection criteria:**

randomized controlled trials (RCTs) and intervention studies of community-dwelling adults aged ≥50 years or with a mean age of >60 years that assessed the effect of a cardiovascular assessment and interventions in reducing fall risk. Key search concepts were “falls” and “aged”, and terms for different cardiovascular evaluations and interventions were included. The Cochrane Checklist for risk of bias and the ROBINS-I tool were used to assess the quality of the studies.

**Results:**

seven studies were included. The majority showed a reduction in falls after cardiovascular evaluation and intervention. Two out of four studies that focused on carotid sinus hypersensitivity (CSH) as a modifiable cardiovascular risk factor for falls, showed a significant reduction in falls after pacemaker implantation. Two studies that looked at sinus node dysfunction (SND) both showed a significant reduction in falls after pacemaker implantation. One study showed that 33% of the patients experienced a fall after cardiovascular evaluation and intervention, whereas all patients fell before assessment.

**Conclusions:**

the majority of the included studies showed a reduction in falls after the intervention. However, the number of published papers regarding the effect of cardiovascular assessment and interventions on falls is small. A standardized assessment of cardiovascular risk factors may be essential in preventing falls in older adults and could consequently reduce injuries, loss of quality of life, deaths, and fall-related expenditures.

## Background

Falls among older people are a major problem, as one in three people of 65 years or older falls each year (1). Falls can lead to injuries, reduction in quality of life and even death (2, 3) and are responsible for at least 25 billion euros in healthcare costs yearly in the European Union (4). Since the number of fall-related hospital admissions is expected to rise by 50% by 2030 (5), this drastic increase in accompanying health care consumption should be anticipated. Therefore, it is of great importance to identify treatable factors in order to prevent falls and by that reduce fall-related expenditures.

Multiple risk factors associated with falling have been identified, including age, gender, impaired balance and gait, medication, and cardiovascular diseases (6). The latter may contribute to a fall by inducing cerebral hypoperfusion, resulting in dizziness, temporary loss of consciousness or falls (7). Furthermore, there is increasing evidence that in patients with cardiovascular diseases such as atrial fibrillation, cerebral white matter lesions contribute to fall risk through mobility disorders and cognitive and mood impairment (8, 9, 10). A systematic review from our group (2016) showed strong associations between cardiovascular disorders and falls (11). The most consistent associations with falls were observed for low blood pressure, heart failure and cardiac arrhythmia and specifically atrial fibrillation. A higher prevalence of carotid sinus hypersensitivity, vasovagal collapse, and postprandial hypotension was seen in fallers. Coronary artery disease, orthostatic hypotension, general cardiovascular disease and hypertension showed inconsistent associations with falls. Finally, arterial stiffness and several echocardiographic abnormalities were associated with falls (single studies). Since then, several studies have endorsed the association between fall risk and orthostatic hypotension, structural cardias abnormalities, and atrial fibrillation as well as other arrhythmias (11, 12, 13, 14, 15, 16, 17). These cardiovascular disorders form potentially modifiable risk factors for falls.

With regard to echocardiographic and electrocardiographic abnormalities, recent studies have strengthened the earlier findings linking atrial fibrillation, poor left ventricular function, and valve abnormalities to falls (16, 17, 18, 19, 20, 21, 22). This suggests that greater awareness of atrial fibrillation and structural cardiac abnormalities may be necessary in older fallers. Thus, in order to reveal cardiovascular fall risk factors, a thorough cardiovascular evaluation is essential. This evaluation usually starts with detailed history taking and physical examination, potentially supplemented with an electrocardiogram (ECG), echocardiography, carotid sinus massage, tilt-table testing and/or other cardiovascular testing, depending on the initial findings. Current guidelines underline the significance of a cardiovascular assessment in the evaluation of fallers (23), but in current medical practice this is not yet routinely performed (24).

Although the association between cardiovascular abnormalities and fall risk is clear, little research has been done to assess the effect of cardiovascular interventions on fall risk (25). The aim of this scoping review is to explore the current literature on the effectiveness of single and comprehensive cardiovascular evaluations and interventions in reducing fall risk in older people.

## Methods

To answer the research question we conducted a scoping review according to the framework outlined by Levac (26), adapted from Arksey and O’Malley (27). The framework consists of five stages in which the first stage refers to identifying the research question (see above). The other consecutive stages subsequently address the identification of relevant studies, study selection, data charting, and summarizing and reporting the results.

### Stage 2: Identifying relevant studies

With help of a clinical librarian (JD), we systematically searched Medline and Cochrane Library from onset until June 28th 2018 for studies about effectiveness of cardiovascular evaluations and interventions on fall risk. We also searched the WHO ICTRP Search Portal for unpublished studies. Key search concept combinations were [persons 50 years or older] AND [cardiovascular evaluation OR cardiovascular interventions] AND [falls]. Search terms for cardiovascular evaluations included blood pressure measurements, tilt-table test, carotid sinus massage, electrocardiogram (ECG), echocardiography, Holter monitoring and loop recorder. Search terms for cardiovascular interventions included pacemaker, cardiac valve replacement, coronary angioplasty and catheter ablation. The complete search strategy is provided as a supplement in Appendix I.

### Stage 3: Study selection

We included all randomized controlled trials and intervention studies of community-dwelling adults aged ≥50 years or with a mean age of >60 years that looked at falls as an outcome measure, and that assessed the effect of a cardiovascular assessment and intervention. Interventions could comprise either multifactorial or single cardiovascular interventions. Studies with hospitalized or other noncommunity- dwelling participants were also included. Articles were excluded if they were reviews or conference abstracts, if they were not written in English, if the intervention was not clearly defined, or if they applied to a specific subgroup (e.g. patients with Parkinson’s disease).

The articles found were selected for inclusion independently by two reviewers (SJ and SL) by screening titles and abstracts. Discrepancies in article inclusion were solved by consulting a third reviewer (NV). The two reviewers (SJ and SL) independently assessed all full-text articles for eligibility.

### Stage 4: Charting the data

To address the research question, a data charting form was developed with relevant variables, including: author, year, design, sample size, (mean) age of participants, setting, intervention, control, duration of follow-up, outcome of falls, results, and conclusions. The two reviewers (SJ and SL) independently extracted data from all included studies using this charting form. Discrepancies between results were resolved through discussion. If discrepancies remained unsolved, the third reviewer (NV) was consulted.

### Stage 5: Collating, summarizing, and reporting the results

The data found was collated and summarized in a descriptive table (Table 1). To assess quality of the included studies the Cochrane Checklist for risk of bias was used for RCTs (28), and the ROBINS-I tool for included intervention studies (29). Details of the quality assessment are described in Appendix II.

**Table 1:** Overview of studies published on cardiovascular evaluation/interventions and falls

**Studies**	**Study population Design**	**N**	**Mean age (SD)**	**Gender (% female)**	**Setting**	**Population**	**Intervention**	**Exposure Control**	**Duration of follow-up (mean)**	**Outcome Outcome of falls**	**Results**	**Conclusion**
Brenner, 2017 (35)	Non-randomized, prospective multicentre study	78	75.4 (8.3)	50.6	Cardiology department	Patients with SND (based on 12-lead or Holter ECG) and a class I indication for PPM implantation	Dual-chamber pacemaker	None	12 months	Primary: fall-incidence Secondary: number of falls (with or without injury/requiring medical treatment/fracture) Fall data before PM implantation retrospectively collected; after PM implantation prospectively.	53% of patients fell before implantation vs. 15% after implantation (p<0.001, RRR 71%) 127 falls before implantation vs. 13 falls after implantation (p<0.001, RRR 90%). 28% of patients had falls with injury before implantation vs. 10% after implantation (p=0.005, RRR 63%), 31% had falls requiring medical treatment vs. 8% after implantation (p<0.001, RRR 75%), 8% had falls with fracture after implantation vs. 0% after implantation (p=0.013).	Permanent pacemaker implantation is associated with a significantly reduced fall-incidence, fall-related injuries and fractures in patients with SND.
Crilley, 1997 (30)	Non-randomized, prospective study	37	79.0 (range 58-91)	57.1	Geriatric department	Patients with recurrent falls, dizziness, syncope, and a hypersensitive cardio inhibitory reflex (based on CSM and/or HUTT)	Dual-chamber pacemaker	None	10 months (range 1.5-30)	Fall-incidence. Fall data retrospectively collected.	81% of patients had falls before implantation vs. 30% after implantation (p<0.0001)	Fall-incidence in patients with CSH was significantly reduced after pacemaker implantation.
Jansen, 2015 (36)	Non-randomized, prospective singlecentre study	15	74.6 (6.6)	66.7	Falls clinic	Patients with one or more falls in the past year who presented in the ER or were referred	Comprehensive cardiovascular assessment, including structured history, echocardiography, ECG and tilt-table testing, followed by a multidisciplinary evaluation and treatment advice	None	6 months	Primary: identification of a new cardiovascular diagnosis contributing to a fall Secondary: Number of falls Fall data prospectively collected.	47% of patients were diagnosed with a cardiovascular abnormality contributing to a fall. 33% of patients had a fall incident during follow-up. CVDs contributing to a fall were: initial and delayed OH, drug induced hypotension and carotid sinus syndrome	A comprehensive cardiovascular assessment in fallers led to the identification of cardiovascular abnormalities contributing to falls in almost half of participants.
Kenny, 2001 (31)	Single-centre RCT	159	73.0 (10)	59.4	Emergency department and cardiovascular investigation unit	Non-accidental fallers with cardio inhibitory or mixed CSH (based on CSM)	Dual-chamber pacemaker	Standard treatment (not specified)	12 months	Number of falls. Number of injurious events. Fall data prospectively collected.	OR 0.42 (0.23-0.75) of falling with pacemaker vs. controls. OR 0.42 (0.31-0.57) of falling with pacemaker vs. controls. 88 controls reported 669 falls (mean 9.3; range 0 to 89), and 87 paced patients reported 216 falls (mean 4.1; range 0 to 29) Injurious events reduced by 70% (202 in controls vs. 61 events in paced patients).	Fall-incidence in patients with CSH was significantly reduced after pacemaker implantation.
Krasniqi, 2012 (34)	Non-randomized, prospective singlecentre study	124	71.9 (9.7)	39.3	Tertiary cardiology clinic	Patients with SND (based on Holter ECG) that underwent PPM implantation	Pacemaker (not specified)	None	>12 months (mean 2.3 years)	Number of (injured) fallers Number of falls (with injury) Fall data retrospectively collected.	32.3% of patients fell before PM implantation vs. 8.1% after PM implantation (p<0.001, RRR 74.9%). 15.3% of patients had a fall with injury before PM implantation vs. 5.6% after PM implantation (p=0.014, RRR 63.4%) Number of falls reduced from 60 to 22 after PM implantation (p=0.035, RRR 63.3%). Number of falls with injury reduced from 22 to 7 after PM implantation (p=0.013, RRR 66.7%)	Pacemaker implantation significantly reduced number of fallers, number of falls, and fall related injuries in patients with SND.
Parry, 2009 (32)	Single-centre randomized, double-blind, crossover, placebo controlled trial	25	76.8 (9)	79.4	Emergency department or specialist falls and syncope facility	Patients with three or more unexplained falls who had CICSH or mixed CSH based on CSM	Dual-chamber permanent pacemaker (turned on)	Dual-chamber permanent pacemaker (turned off)	12 months	Primary: number of falls Secondary: time to first fall Fall data prospectively collected.	Pacing intervention had no effect on number of falls (Mean of 4.04 falls (9.54) in paced mode, and 3.48 (7.22) falls) in placebo mode). RR 0.82 (0.62-1.10) of falling with pacemaker turned on vs. pacemaker turned off.	Permanent pacing in older fallers with CSH had no effect on fall incidence.
Ryan, 2010 (33)	Multicentre, doubleblind, RCT	128	78.0 (7)	61.7	Syncope unit	Patients with two or more unexplained falls and/or one syncopal event in the previous year, and CICSH based on CSM	Dual-chamber pacemaker	Implantable loop recorder	24 months	Primary: number of falls Secondary: time to first fall Fall data prospectively collected.	RR 0.79 (0.41-1.50) of falling with pacemaker vs. loop recorder. RR 0.23 (0.15-0.32) of falling after device implantation vs. before. HR 1.34 (0.84-2.12) of falling with pacemaker vs. loop recorder	There was no difference in fall incidence between patients with CSH who were paced and who were not.


(CI)CSH: (cardio inhibitory) carotid sinus hypersensitivity | CSM: carotid sinus massage | ER: emergency room | HR: hazard ratio | HUTT: head-up tilt test | OH: orthostatic hypotension | OR: odds ratio | (P)PM: (permanent) pacemaker | RCT: randomized controlledtrial | RR(R): relative risk (reduction)| SND: sinus node dysfunction

## Results

### Search Result and Study Characteristics

**Figure 1:**
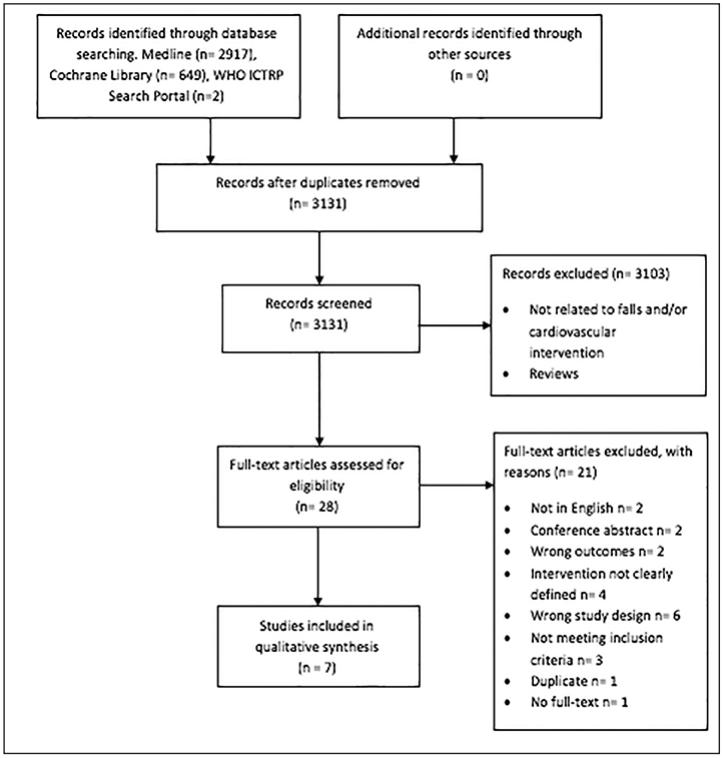
Flow diagram of screened and included studies

Our initial search retrieved a total of 3131 studies; of 28 papers the full text was reviewed. Seven articles were included in this review. Figure 1 shows the search and selection process. Table 1 summarizes the findings relevant to the research question. Four studies selected fallers with carotid sinus hypersensitivity (CSH) (30, 31, 32, 33), two studies selected fallers with sinus node dysfunction (SND) (34, 35), and one study selected geriatric patients from a specialized falls clinic. Three studies were randomized controlled trials (31, 32, 33), four were non-randomized intervention studies of which one was a pilot study (30, 31, 32, 33, 34). The number of participants varied between 15 and 159 and mean age varied from 71.9 to 79.0 years. Two studies were conducted at a cardiology department (34, 35), two in a syncope unit (32, 33), one at a geriatric department (30), one at a specialized falls clinic (36), and one at an emergency department (31). All studies that were carried out in hospital included patients that had presented with falls from the community. Fall data were collected prospectively in four studies (31-33, 36), and retrospectively in two studies (30, 34). One study collected fall data retrospectively before intervention and prospectively after intervention (35).

### Carotid Sinus Hypersensitivity (CSH)

Effectiveness of cardiovascular evaluations and interventions on fall risk in patients with CSH was assessed in four studies, of which three were RCTs and one was an intervention study (30, 31, 32, 33). Two studies were conducted at a syncope unit, one at a geriatric department, and one at an emergency department/cardiovascular investigation unit. All studies defined CSH as at least asystole induced by carotid sinus massage (CSM). Additionally performed cardiovascular evaluations to determine the eligibility for cardiac pacing were: ECG, head-up tilt table testing, echocardiogram, cardiac electrophysiology, blood pressure monitoring, Holter ECG, routine blood screen, and/or orthostatic blood pressure measurement. All four studies investigated the effect of a dual-chamber pacemaker on fall risk in patients with unexplained falls, of which two reported a reduction in falls.

First, the RCT conducted by Kenny et al. showed an odds ratio (OR) of 0.42 (95% confidence interval (CI) 0.23-0.75) of falling with pacemaker compared with controls. Injurious events were reduced by 70% in paced patients (31). Second, the non-randomized study by Crilley et al. showed that 81% of patients with CSH had falls in the year before implantation, compared with 30% after implantation (30). The other two studies showed no effect of pacemaker implantation. The multicenter RCT of Ryan et al. showed a risk ratio (RR) of 0.79 (95% CI 0.41-1.50) of falling with pacemaker implantation compared with loop recorder implantation (33). The cross-over RCT of Parry et al. showed a RR of 0.82 (95% CI 0.62-1.10) of falling with pacemaker turned on compared with pacemaker turned off (32). All four studies had a low or moderate risk of bias according to the Cochrane Checklist and ROBINS-I tool (Table 2 and Table 3).

**Table 2:** Quality assessment of non-randomized studies (ROBINS-I Checklist)

	Kenny, 2001	Parry, 2009	Ryan, 2010
Was the allocation of the intervention to the patients randomized?	?*	+	+
The person who includes patients should not be aware of the randomization sequence. Was that the case here?	?	+	?
Were the patients and the practitioners blinded for the treatment?	-	+	+
Were the effect assessors blinded for treatment?	?	?	?
Were the groups comparable at the beginning of the trial? If not: has this been corrected in the analyzes?	+	+†	+
Is a complete follow-up available from a sufficient proportion of all participants? If not: is selective loss-to-follow-up sufficiently excluded?	+	−‡	+
Have all the included patients been analyzed in the group in which they were randomized?	+	+	-
Have the groups been treated equally, apart from the intervention?	?	+	?
Is selective publication of results sufficiently excluded?	?	?	?
Is unwanted influence of sponsors sufficiently excluded?	?	+	+


* block randomization, in blocks of eight; † Crossover study; ‡ >20% dropout

**Table 3:** Quality assessment of non-randomized studies (ROBINS-I Checklist)

	**Brenner, 2017**	**Crilley, 1997**	**Jansen, 2015**	**Krasniqi, 2012**
Bias due to confounding	Low	Serious	Moderate	Serious
Bias in selection of participants into the study	Low	Low	Moderate	Serious
Bias in classification of interventions	Low	Low	Low	Moderate
Bias due to deviations from intended interventions	Low	Low	Low	Low
Bias due to missing data	Moderate	Serious	Low	Moderate
Bias in measurement of outcomes	Moderate	Moderate	Moderate	Moderate
Bias in selection of the reported result	Low	Low	Low	Low
Overall bias	Low	Moderate	Low	Serious

### Sinus Node Dysfunction (SND)

Two studies studied the effect of pacemaker implantation on fall risk in patients with SND (34, 35). Both studies were non-randomized intervention studies and selected cardiology referrals with SND based on either symptoms compatible with SND, 12-lead ECG and/or Holter ECG assessment. All included patients had a pacemaker implanted, and fall rates were assessed after at least 12 months of follow-up. The two studies reported a significant decrease in fall rates, total number of falls, and fall-related injuries in patients with a pacemaker implanted. The study of Brenner et al. had a low risk of bias according to the ROBINS-I tool. The study of Krasniqi et al. had a serious risk of bias according to the ROBINS-I tool due to bias of selection of participants and bias due to confounding (Table 3).

### Comprehensive Cardiovascular Assessment

Jansen et al. carried out a comprehensive cardiovascular assessment and subsequent cardiovascular intervention in their small pilot study (36). Almost half of older fallers had a treatable cardiovascular abnormality contributing to fall risk of the individual patient, and a cardiovascular evaluation with subsequent intervention could identify these abnormalities. Furthermore, 33% of the patients experienced a fall after cardiovascular evaluation and intervention, whereas all patients fell before assessment. The study had a low risk of bias according to the ROBINS-I tool (Table 3).

## Discussion

This scoping review shows that the majority of studies that investigated the effect of a cardiovascular assessment with subsequent intervention in older fallers showed a reduction in falls after the intervention. Main cardiovascular risk factors for falls focused on in the included studies were CSH and SND. These cardiovascular abnormalities are associated with syncope and falls in older people (37, 38). However, this review of current literature shows that evidence about the effectiveness of cardiovascular assessment and subsequent interventions on fall risk is scarce, and that most studies have only focused on single cardiovascular fall risk increasing factors.

Four studies focused on CSH as a modifiable cardiovascular risk factor for falls, and half of them showed a significant reduction in falls after pacemaker implantation. Kenny et al. showed that falls and injurious events were less common in subjects with CSH after pacemaker implantation. Crilley et al. also showed that patients with CSH had less falls after pacemaker implantation. However, the study of Crilley et al. collected fall-data retrospectively which is a potential risk of bias. The other two studies that assessed the effect of pacemaker implantation in patients with CSH showed no effect of pacing on fall risk, although the study of Parry et al. might be underpowered due to a high drop-out rate.

The two studies that looked at SND both showed a significant reduction in falls after pacemaker implantation. However, although the outcome of these studies was falls, the studies primarily included patients with established SND, and information on falls before pacemaker implantation was recorded retrospectively. Furthermore, these studies lacked a control group, so a causal relationship cannot be established.

The pilot study of Jansen et al. is the only study that has broadly assessed modifiable cardiovascular fall risk factors. They showed that adding a standardized cardiovascular assessment and intervention including structured history taking to the multifactorial falls evaluation led to the identification of cardiovascular abnormalities contributing to fall risk in 47% of older fallers. Furthermore, Jansen et al. found that 33% of the patients experienced a fall after cardiovascular evaluation and intervention, whereas all patients fell before assessment [36]. However, being a pilot study, the study primarily assessed feasibility and not effects on fall risk. Thus it comprised a small group of participants and lacked a control group.

Besides the studies in our review, we found a study that assessed the effect of a cardiovascular intervention on fall risk, but they lacked the appropriate outcome to meet the inclusion criteria of this review. Tarro Genta et al. evaluated a cardiac rehabilitation program in patients that underwent transcatheter aortic valve implantation (TAVI) compared with patients that had surgical aortic valve replacement (sAVR). Fall risk was measured with the Morse Fall Scale (MFS), and the results showed that fall risk on admission and at discharge was worse in patients who underwent TAVI, compared with patients that underwent sAVR (39). However, this study was not an RCT and patients that underwent TAVI had more risk factors for falling at baseline (e.g. older, higher proportion of coronary heart disease, more comorbidity, lower left ventricular systolic function). Moreover, TAVI-patients were more disabled according to the Barthel-index. Thus, a causal relationship between TAVI, sAVR and fall risk could not be established with this study.

As mentioned above, up to now little research has been performed on the effectiveness of cardiovascular evaluation and interventions on fall risk, despite the evidence of cardiovascular risk factors for falling (11). Several studies that looked at multifactorial falls interventions have included cardiovascular assessments and interventions. Gobierno Hernandez et al. included referral to a cardiologist as part of their multifactorial intervention and found no significant reduction of falls (40). Tan et al. included a comprehensive cardiovascular assessment and interventions as part of their multifactorial intervention and also did not find a difference in fall rate compared with controls (41). Rubenstein et al. included ECG and 24-hour Holter monitoring in their intervention group and found 9% less falls in the intervention group, but this was not statistically significant (42). Lightbody et al. evaluated a multifactorial fall prevention program including a cardiovascular assessment with ECG and blood pressure measurement and reported fewer falls in the intervention group compared with controls, although not significant (43). Due to the multifactorial nature of these trials, the contribution of the cardiovascular assessment to the reduction of falls cannot be established. Moreover, the cardiovascular components of these study-assessments were often very limited. However, Tinetti et al. showed that participants in a multifactorial intervention group had significantly fewer falls compared with controls, and that improvement in postural blood pressure change was partly responsible for this reduction in falls (44).

As syncope and symptoms of falls overlap, syncope in older persons is often mistaken for falls (45). Recent research on falls and syncope in older persons has shown that a multidisciplinary comprehensive assessment at a falls and syncope clinic consisting of a 12-lead ECG, blood pressure measurements (supine and active standing), echocardiogram, 24-hour Holter monitoring, and tilt testing with carotid sinus massage (on indication) could identify possible causes for falls and syncope in 94% of the patients (20). Eighty-three percent of the patients were diagnosed with hypotension, 44% of the patients had a cardiac cause for syncope, and 21% had reflex syncope. Only in 6% of the patients the cause of syncope remained unexplained. Remarkably, 50% of the syncope patients in this study presented with falls only, thus underlining the need of a cardiovascular assessment in older fallers. Moreover, Zwart et al. found that atrial fibrillation (AF) prevalence is underestimated in the geriatric population, and that an additional 50% of AF cases were found by using 24-hour Holter ECG in comparison to a 12-lead ECG and medical history taking (22). The results of De Ruiter et al., Zwart et al., and the pilot study of Jansen et al., showed that cardiovascular causes for falls (and syncope) can be easily overlooked. A comprehensive standardized cardiovascular assessment will likely contribute to recognition of these cardiovascular causes, thereby presenting modifiable factors to reduce fall risk in older persons. However, it is important to remark that these additional tests should always be preceded by detailed history taking and physical examination as this remains the cornerstone in identifying cardiovascular risk factors for syncope or falls (46, 47, 48, 49).

Limitations of our review are the limited number of studies. Also, the differences in study populations make it difficult to compare studies and draw conclusions. A meta-analysis could not be performed because of the large heterogeneity of studies.

## Conclusion

There is clear evidence that cardiovascular abnormalities are associated with increased fall risk in older persons. However, only few studies have investigated whether cardiovascular evaluations and interventions reduce falls, even though an increasing body of evidence shows that cardiovascular causes of falls are often underestimated. Furthermore, most studies have included only single cardiovascular interventions. This underlines the need for a well-designed randomized controlled trial to evaluate the efficacy of a broad cardiovascular evaluation and subsequent intervention in reducing falls. Additional testing should always be preceded by detailed and structured history taking and physical examination to identify cardiovascular risk factors for syncope or falls. A standardized assessment of cardiovascular risk factors with subsequent additional testing and accompanying interventions may be essential in preventing falls in older adults, and could consequently reduce injuries, loss of quality of life, deaths, and fall-related expenditures.

## Electronic supplementary material

Supplementary material, approximately 44 KB.
