# Supplementary material for: Effectiveness of Cardiovascular Evaluations and Interventions on Fall Risk: A Scoping Review
Source: J Nutr Health Aging. 2019 Feb 7;23(4):330–7. doi: 10.1007/s12603-019-1165-2 (PMC6507528; doi:10.1007/s12603-019-1165-2)
Supplement: Supplementary file 1 — Supplementary material, approximately 44 KB. [file mmc1.docx]

**APPENDIX I. Search strategy**

**Ovid MEDLINE(R) Epub Ahead of Print, In-Process & Other Non-Indexed Citations, Ovid MEDLINE(R) Daily and Ovid MEDLINE(R) <1946 to 28 June 2018> (2917 hits)**

1 Geriatric assessment/ or frail elderly/ or exp aged/ or middle aged/ or exp nursing homes/ or "homes for the aged"/

2 (older person? or older patient? or middle aged or seniors or senior citiz* or elder or elders or elderly or geriatric* or frailty or postmenopausal women or community-dwelling or nursing home? or resident* or old* people or old* person? or old* patient? or old* client? or old* adult?).ab,kf,ti.

3 (geriatr* or age or aging or elderl*).in,jw.

4 or/1-3 [persons 50 yrs or older]

5 ((cardiovascular adj3 (assessment or evaluation? or intervention?)) or (Blood Pressure adj3 (monitoring or self or ambulatory or home))).ab,kf,ti.

6 (Orthostatic hypotension or postural hypotension or Tilt-table test or head-down tilt or Carotid sinus massage or Electrocardiogram or ECG or Electrocardiograph or Transthoracic Echocardiography or Cross-Sectional Echocardiography or Cross Sectional Echocardiography or "m-Mode" or Contrast Echocardiography or 2D Echocardiography or Two Dimensional Echocardiography or "2-D Echocardiography" or Dynamic Electrocardiography or Ambulatory Electrocardiographic Monitoring or Ambulatory Electrocardiography or Holter Electrocardiography or Holter Monitoring or Loop recorder).ab,kf,ti.

7 exp angioplasty/

8 (Transcatheter Aortic Valve Replacement or Transcatheter Aortic Valve Implantation or Cardiac valve replacement or Myocardial Revascularization or coronary Atherectomy or Coronary Artery Bypass or Angioplast* or Endoluminal Repair? or Transluminal Arterial Dilation? or catheter Ablation or Transvenous Electric* Ablation or Radiofrequency Catheter Ablation or pacemaker? or Cardiac Valve Annuloplast* or Valvular Annuloplasty or Valvular Annuloplast* or Heart Valve Annuloplasty or Cardiac Valve Annulus Repair or heart Valve Annulus Repair or Cardiac Valve Annular Repair or Heart Valve Annular Repair or Cardiac Valve Annular Reduction or Cardiac Valve Annulus Shortening or Cardiac Valve Annulus Reduction or Mitral Valve Annuloplast* or Mitral Valve Annulus Repair or (Coronary adj3 Balloon) or Coronary Angioplasty).ab,kf,ti.

9 or/5-8 [cardiovascular evaluation | -interventions]

10 accidental falls/

11 (fall? or fell or falling or fallen or faller or stumble? or stumbling or stumbles or slip or slips or slipping or slipped or trip or tripped).ab,kf,ti.

12 10 or 11

13 and/4,9,12

14 (ISRCTN11674947 or NCT01037426 or myfait or (safe pace and (study or trial))).ab,kf,ti.

15 13 or 14

16 remove duplicates from 15

**Cochrane Library (Wiley Online Library), Search date: 28 June 2018 (649 hits)**

#1 ("homes for the aged" or older person? or older patient? or middle aged or seniors or senior citiz* or elder or elders or elderly or geriatric* or frailty or postmenopausal women or community-dwelling or nursing home? or resident* or old* people or old* person? or old* patient? or old* client? or old* adult?):ti,ab,kw (Word variations have been searched)

#2 ((cardiovascular near/2 (assessment or evaluation? or intervention?)) or (Blood Pressure near/2 (monitoring or self or ambulatory or home))):ti,ab,kw (Word variations have been searched)

#3 (Orthostatic hypotension or postural hypotension or Tilt-table test or head-down tilt or Carotid sinus massage or Electrocardiogram or ECG or Electrocardiograph or Transthoracic Echocardiography or Cross-Sectional Echocardiography or Cross Sectional Echocardiography or "m-Mode" or Contrast Echocardiography or 2D Echocardiography or Two Dimensional Echocardiography or "2-D Echocardiography" or Dynamic Electrocardiography or Ambulatory Electrocardiographic Monitoring or Ambulatory Electrocardiography or Holter Electrocardiography or Holter Monitoring or Loop recorder):ti,ab,kw (Word variations have been searched)

#4 (Transcatheter Aortic Valve Replacement or Transcatheter Aortic Valve Implantation or Cardiac valve replacement or Myocardial Revascularization or coronary Atherectomy or Coronary Artery Bypass or Angioplast* or Endoluminal Repair? or Transluminal Arterial Dilation? or catheter Ablation or Transvenous Electric* Ablation or Radiofrequency Catheter Ablation or pacemaker? or Cardiac Valve Annuloplast* or Valvular Annuloplasty or Valvular Annuloplast* or Heart Valve Annuloplasty or Cardiac Valve Annulus Repair or heart Valve Annulus Repair or Cardiac Valve Annular Repair or Heart Valve Annular Repair or Cardiac Valve Annular Reduction or Cardiac Valve Annulus Shortening or Cardiac Valve Annulus Reduction or Mitral Valve Annuloplast* or Mitral Valve Annulus Repair or (Coronary near/2 Balloon) or Coronary Angioplasty):ti,ab,kw (Word variations have been searched)

#5 #2 or #3 or #4

#6 (fall? or fell or falling or fallen or faller or stumble? or stumbling or stumbles or slip or slips or slipping or slipped or trip or tripped):ti,ab,kw (Word variations have been searched)

#7 #1 and #5 and #6

#8 ISRCTN11674947 or NCT01037426 (Word variations have been searched)

#9 myfait or ("safe pace" and (study or trial)):ti,ab,kw (Word variations have been searched)

#10 #7 or #8 or

**APPENDIX II. Quality Assessment**

**Risk of bias assessment Brenner et al. 2017**

Responses underlined in green are potential markers for low risk of bias, and responses in red are potential markers for a risk of bias. Where questions relate only to sign posts to other questions, no formatting is used.

|  | **Signalling questions** | | **Description** | **Response options** |
| --- | --- | --- | --- | --- |
| **Bias due to confounding** | | | | |
|  | 1.1 Is there potential for confounding of the effect of intervention in this study?  **If N/PN to 1.1:** the study can be considered to be at low risk of bias due to confounding and no further signalling questions need be considered | |  | PY |
|  | **If Y/PY to 1.1**: determine whether there is a need to assess time-varying confounding: | |  |  |
|  | 1.2. Was the analysis based on splitting participants’ follow up time according to intervention received?  **If N/PN**, answer questions relating to baseline confounding (1.4 to 1.6)  **If Y/PY**, go to question 1.3. | |  | N |
|  | 1.3. Were intervention discontinuations or switches likely to be related to factors that are prognostic for the outcome?  **If N/PN**, answer questions relating to baseline confounding (1.4 to 1.6)  **If Y/PY**, answer questions relating to both baseline and time-varying confounding (1.7 and 1.8) | |  | NA / Y / PY / PN / N / NI |
|  | **Questions relating to baseline confounding only** | | | |
|  | 1.4. Did the authors use an appropriate analysis method that controlled for all the important confounding domains? |  | Y | |
|  | 1.5. **If Y/PY to 1.4**: Were confounding domains that were controlled for measured validly and reliably by the variables available in this study? |  | Y | |
|  | 1.6. Did the authors control for any post-intervention variables that could have been affected by the intervention? |  | NA | |
|  | **Questions relating to baseline and time-varying confounding** | |  | |
|  | 1.7. Did the authors use an appropriate analysis method that controlled for all the important confounding domains and for time-varying confounding? |  | PY | |
|  | 1.8. **If Y/PY to 1.7**: Were confounding domains that were controlled for measured validly and reliably by the variables available in this study? |  | NA | |
|  | **Risk of bias judgement** |  | Low | |
|  | Optional: What is the predicted direction of bias due to confounding? |  | Favours experimental / Favours comparator / Unpredictable | |

| **Bias in selection of participants into the study** | | | |
| --- | --- | --- | --- |
|  | 2.1. Was selection of participants into the study (or into the analysis) based on participant characteristics observed after the start of intervention?  **If N/PN to 2.1:** go to 2.4 |  | N |
|  | 2.2. **If Y/PY to 2.1**: Were the post-intervention variables that influenced selection likely to be associated with intervention?  2.3 **If Y/PY to 2.2**: Were the post-intervention variables that influenced selection likely to be influenced by the outcome or a cause of the outcome? |  | NA / Y / PY / PN / N / NI  NA / Y / PY / PN / N / NI |
|  | 2.4. Do start of follow-up and start of intervention coincide for most participants? |  | Y |
|  | 2.5. **If Y/PY to 2.2 and 2.3, or N/PN to 2.4**: Were adjustment techniques used that are likely to correct for the presence of selection biases? |  | NA / Y / PY / PN / N / NI |
|  | **Risk of bias judgement** |  | Low |
|  | Optional: What is the predicted direction of bias due to selection of participants into the study? |  | Favours experimental / Favours comparator / Towards null /Away from null / Unpredictable |

| **Bias in classification of interventions** | | | |
| --- | --- | --- | --- |
|  | 3.1 Were intervention groups clearly defined? |  | Y |
|  | 3.2 Was the information used to define intervention groups recorded at the start of the intervention? |  | Y |
|  | 3.3 Could classification of intervention status have been affected by knowledge of the outcome or risk of the outcome? |  | PN |
|  | **Risk of bias judgement** |  | Low |
|  | Optional: What is the predicted direction of bias due to classification of interventions? |  | Favours experimental / Favours comparator / Towards null /Away from null / Unpredictable |

| **Bias due to deviations from intended interventions** | | | |
| --- | --- | --- | --- |
|  | **If your aim for this study is to assess the effect of assignment to intervention, answer questions 4.1 and 4.2** | |  |
|  | 4.1. Were there deviations from the intended intervention beyond what would be expected in usual practice? |  | N |
|  | 4.2. **If Y/PY to 4.1**: Were these deviations from intended intervention unbalanced between groups *and* likely to have affected the outcome? |  | NA / Y / PY / PN / N / NI |
|  | **If your aim for this study is to assess the effect of starting and adhering to intervention, answer questions 4.3 to 4.6** | |  |
|  | 4.3. Were important co-interventions balanced across intervention groups? |  | Y / PY / PN / N / NI |
|  | 4.4. Was the intervention implemented successfully for most participants? |  | Y / PY / PN / N / NI |
|  | 4.5. Did study participants adhere to the assigned intervention regimen? |  | Y / PY / PN / N / NI |
|  | 4.6. **If N/PN to 4.3, 4.4 or 4.5**: Was an appropriate analysis used to estimate the effect of starting and adhering to the intervention? |  | NA / Y / PY / PN / N / NI |
|  | **Risk of bias judgement** |  | Low |
|  | Optional: What is the predicted direction of bias due to deviations from the intended interventions? |  |  |

| **Bias due to missing data** | | | |
| --- | --- | --- | --- |
|  | 5.1 Were outcome data available for all, or nearly all, participants? |  | Y |
|  | 5.2 Were participants excluded due to missing data on intervention status? |  | Y |
|  | 5.3 Were participants excluded due to missing data on other variables needed for the analysis? |  | PN |
|  | 5.4 **If PN/N to 5.1, or Y/PY to 5.2 or 5.3**: Are the proportion of participants and reasons for missing data similar across interventions? |  | NA |
|  | 5.5 **If PN/N to 5.1, or Y/PY to 5.2 or 5.3**: Is there evidence that results were robust to the presence of missing data? |  | PY |
|  | **Risk of bias judgement** |  | Low / Moderate |
|  | Optional: What is the predicted direction of bias due to missing data? |  | Favours experimental / Favours comparator / Towards null /Away from null / Unpredictable |

| **Bias in measurement of outcomes** | | | |
| --- | --- | --- | --- |
|  | 6.1 Could the outcome measure have been influenced by knowledge of the intervention received? |  | PN |
|  | 6.2 Were outcome assessors aware of the intervention received by study participants? |  | PY |
|  | 6.3 Were the methods of outcome assessment comparable across intervention groups? |  | PY |
|  | 6.4 Were any systematic errors in measurement of the outcome related to intervention received? |  | NI |
|  | **Risk of bias judgement** |  | Low / Moderate |
|  | Optional: What is the predicted direction of bias due to measurement of outcomes? |  | Favours experimental / Favours comparator / Towards null /Away from null / Unpredictable |

| **Bias in selection of the reported result** | | | |
| --- | --- | --- | --- |
|  | Is the reported effect estimate likely to be selected, on the basis of the results, from... |  |  |
|  | 7.1. ... multiple outcome *measurements* within the outcome domain? |  | N |
|  | 7.2 ... multiple *analyses* of the intervention-outcome relationship? |  | PN |
|  | 7.3 ... different *subgroups*? |  | N |
|  | **Risk of bias judgement** |  | Low |
|  | Optional: What is the predicted direction of bias due to selection of the reported result? |  | Favours experimental / Favours comparator / Towards null /Away from null / Unpredictable |

| **Overall bias** | | | |
| --- | --- | --- | --- |
|  | **Risk of bias judgement** |  | Low |
|  | Optional: What is the overall predicted direction of bias for this outcome? |  | Favours experimental / Favours comparator / Towards null /Away from null / Unpredictable |

**Risk of bias assessment Crilley et al. 1997**

Responses underlined in green are potential markers for low risk of bias, and responses in red are potential markers for a risk of bias. Where questions relate only to sign posts to other questions, no formatting is used.

|  | **Signalling questions** | | **Description** | **Response options** |
| --- | --- | --- | --- | --- |
| **Bias due to confounding** | | | | |
|  | 1.1 Is there potential for confounding of the effect of intervention in this study?  **If N/PN to 1.1:** the study can be considered to be at low risk of bias due to confounding and no further signalling questions need be considered | |  | Y |
|  | **If Y/PY to 1.1**: determine whether there is a need to assess time-varying confounding: | |  |  |
|  | 1.2. Was the analysis based on splitting participants’ follow up time according to intervention received?  **If N/PN**, answer questions relating to baseline confounding (1.4 to 1.6)  **If Y/PY**, go to question 1.3. | |  | N |
|  | 1.3. Were intervention discontinuations or switches likely to be related to factors that are prognostic for the outcome?  **If N/PN**, answer questions relating to baseline confounding (1.4 to 1.6)  **If Y/PY**, answer questions relating to both baseline and time-varying confounding (1.7 and 1.8) | |  | NA / Y / PY / PN / N / NI |
|  | **Questions relating to baseline confounding only** | | | |
|  | 1.4. Did the authors use an appropriate analysis method that controlled for all the important confounding domains? |  | N | |
|  | 1.5. **If Y/PY to 1.4**: Were confounding domains that were controlled for measured validly and reliably by the variables available in this study? |  | NA / Y / PY / PN / N / NI | |
|  | 1.6. Did the authors control for any post-intervention variables that could have been affected by the intervention? |  | N | |
|  | **Questions relating to baseline and time-varying confounding** | |  | |
|  | 1.7. Did the authors use an appropriate analysis method that controlled for all the important confounding domains and for time-varying confounding? |  | N | |
|  | 1.8. **If Y/PY to 1.7**: Were confounding domains that were controlled for measured validly and reliably by the variables available in this study? |  | NA / Y / PY / PN / N / NI | |
|  | **Risk of bias judgement** |  | Serious | |
|  | Optional: What is the predicted direction of bias due to confounding? |  | Favours experimental / Favours comparator / Unpredictable | |

| **Bias in selection of participants into the study** | | | |
| --- | --- | --- | --- |
|  | 2.1. Was selection of participants into the study (or into the analysis) based on participant characteristics observed after the start of intervention?  **If N/PN to 2.1:** go to 2.4 |  | N |
|  | 2.2. **If Y/PY to 2.1**: Were the post-intervention variables that influenced selection likely to be associated with intervention?  2.3 **If Y/PY to 2.2**: Were the post-intervention variables that influenced selection likely to be influenced by the outcome or a cause of the outcome? |  | NA / Y / PY / PN / N / NI  NA / Y / PY / PN / N / NI |
|  | 2.4. Do start of follow-up and start of intervention coincide for most participants? |  | Y |
|  | 2.5. **If Y/PY to 2.2 and 2.3, or N/PN to 2.4**: Were adjustment techniques used that are likely to correct for the presence of selection biases? |  | NI |
|  | **Risk of bias judgement** |  | Low |
|  | Optional: What is the predicted direction of bias due to selection of participants into the study? |  | Favours experimental / Favours comparator / Towards null /Away from null / Unpredictable |

| **Bias in classification of interventions** | | | |
| --- | --- | --- | --- |
|  | 3.1 Were intervention groups clearly defined? |  | PY |
|  | 3.2 Was the information used to define intervention groups recorded at the start of the intervention? |  | Y |
|  | 3.3 Could classification of intervention status have been affected by knowledge of the outcome or risk of the outcome? |  | N |
|  | **Risk of bias judgement** |  | Low |
|  | Optional: What is the predicted direction of bias due to classification of interventions? |  | Favours experimental / Favours comparator / Towards null /Away from null / Unpredictable |

| **Bias due to deviations from intended interventions** | | | |
| --- | --- | --- | --- |
|  | **If your aim for this study is to assess the effect of assignment to intervention, answer questions 4.1 and 4.2** | |  |
|  | 4.1. Were there deviations from the intended intervention beyond what would be expected in usual practice? |  | PN |
|  | 4.2. **If Y/PY to 4.1**: Were these deviations from intended intervention unbalanced between groups *and* likely to have affected the outcome? |  | NA / Y / PY / PN / N / NI |
|  | **If your aim for this study is to assess the effect of starting and adhering to intervention, answer questions 4.3 to 4.6** | |  |
|  | 4.3. Were important co-interventions balanced across intervention groups? |  | Y / PY / PN / N / NI |
|  | 4.4. Was the intervention implemented successfully for most participants? |  | Y / PY / PN / N / NI |
|  | 4.5. Did study participants adhere to the assigned intervention regimen? |  | Y / PY / PN / N / NI |
|  | 4.6. **If N/PN to 4.3, 4.4 or 4.5**: Was an appropriate analysis used to estimate the effect of starting and adhering to the intervention? |  | NA / Y / PY / PN / N / NI |
|  | **Risk of bias judgement** |  | Low |
|  | Optional: What is the predicted direction of bias due to deviations from the intended interventions? |  |  |

| **Bias due to missing data** | | | |
| --- | --- | --- | --- |
|  | 5.1 Were outcome data available for all, or nearly all, participants? |  | N |
|  | 5.2 Were participants excluded due to missing data on intervention status? |  | Y |
|  | 5.3 Were participants excluded due to missing data on other variables needed for the analysis? |  | NI |
|  | 5.4 **If PN/N to 5.1, or Y/PY to 5.2 or 5.3**: Are the proportion of participants and reasons for missing data similar across interventions? |  | Y |
|  | 5.5 **If PN/N to 5.1, or Y/PY to 5.2 or 5.3**: Is there evidence that results were robust to the presence of missing data? |  | NA |
|  | **Risk of bias judgement** |  | Serious |
|  | Optional: What is the predicted direction of bias due to missing data? |  | Favours experimental / Favours comparator / Towards null /Away from null / Unpredictable |

| **Bias in measurement of outcomes** | | | |
| --- | --- | --- | --- |
|  | 6.1 Could the outcome measure have been influenced by knowledge of the intervention received? |  | PN |
|  | 6.2 Were outcome assessors aware of the intervention received by study participants? |  | PY |
|  | 6.3 Were the methods of outcome assessment comparable across intervention groups? |  | PY |
|  | 6.4 Were any systematic errors in measurement of the outcome related to intervention received? |  | PN |
|  | **Risk of bias judgement** |  | Moderate |
|  | Optional: What is the predicted direction of bias due to measurement of outcomes? |  | Favours experimental / Favours comparator / Towards null /Away from null / Unpredictable |

| **Bias in selection of the reported result** | | | |
| --- | --- | --- | --- |
|  | Is the reported effect estimate likely to be selected, on the basis of the results, from... |  |  |
|  | 7.1. ... multiple outcome *measurements* within the outcome domain? |  | PN |
|  | 7.2 ... multiple *analyses* of the intervention-outcome relationship? |  | PN |
|  | 7.3 ... different *subgroups*? |  | PN |
|  | **Risk of bias judgement** |  | Low |
|  | Optional: What is the predicted direction of bias due to selection of the reported result? |  | Favours experimental / Favours comparator / Towards null /Away from null / Unpredictable |

| **Overall bias** | | | |
| --- | --- | --- | --- |
|  | **Risk of bias judgement** |  | Low / Moderate / Serious / Critical / NI |
|  | Optional: What is the overall predicted direction of bias for this outcome? |  | Favours experimental / Favours comparator / Towards null /Away from null / Unpredictable |

**Risk of bias assessment Jansen et al. 2015**

Responses underlined in green are potential markers for low risk of bias, and responses in red are potential markers for a risk of bias. Where questions relate only to sign posts to other questions, no formatting is used.

|  | **Signalling questions** | | **Description** | **Response options** |
| --- | --- | --- | --- | --- |
| **Bias due to confounding** | | | | |
|  | 1.1 Is there potential for confounding of the effect of intervention in this study?  **If N/PN to 1.1:** the study can be considered to be at low risk of bias due to confounding and no further signalling questions need be considered | |  | Y |
|  | **If Y/PY to 1.1**: determine whether there is a need to assess time-varying confounding: | |  |  |
|  | 1.2. Was the analysis based on splitting participants’ follow up time according to intervention received?  **If N/PN**, answer questions relating to baseline confounding (1.4 to 1.6)  **If Y/PY**, go to question 1.3. | |  | N |
|  | 1.3. Were intervention discontinuations or switches likely to be related to factors that are prognostic for the outcome?  **If N/PN**, answer questions relating to baseline confounding (1.4 to 1.6)  **If Y/PY**, answer questions relating to both baseline and time-varying confounding (1.7 and 1.8) | |  | NA / Y / PY / PN / N / NI |
|  | **Questions relating to baseline confounding only** | | | |
|  | 1.4. Did the authors use an appropriate analysis method that controlled for all the important confounding domains? |  | N | |
|  | 1.5. **If Y/PY to 1.4**: Were confounding domains that were controlled for measured validly and reliably by the variables available in this study? |  | NA / Y / PY / PN / N / NI | |
|  | 1.6. Did the authors control for any post-intervention variables that could have been affected by the intervention? |  | N | |
|  | **Questions relating to baseline and time-varying confounding** | |  | |
|  | 1.7. Did the authors use an appropriate analysis method that controlled for all the important confounding domains and for time-varying confounding? |  | N | |
|  | 1.8. **If Y/PY to 1.7**: Were confounding domains that were controlled for measured validly and reliably by the variables available in this study? |  | NA / Y / PY / PN / N / NI | |
|  | **Risk of bias judgement** |  | Moderate | |
|  | Optional: What is the predicted direction of bias due to confounding? |  | Favours experimental / Favours comparator / Unpredictable | |

| **Bias in selection of participants into the study** | | | |
| --- | --- | --- | --- |
|  | 2.1. Was selection of participants into the study (or into the analysis) based on participant characteristics observed after the start of intervention?  **If N/PN to 2.1:** go to 2.4 |  | N |
|  | 2.2. **If Y/PY to 2.1**: Were the post-intervention variables that influenced selection likely to be associated with intervention?  2.3 **If Y/PY to 2.2**: Were the post-intervention variables that influenced selection likely to be influenced by the outcome or a cause of the outcome? |  | NA / Y / PY / PN / N / NI  NA / Y / PY / PN / N / NI |
|  | 2.4. Do start of follow-up and start of intervention coincide for most participants? |  | N |
|  | 2.5. **If Y/PY to 2.2 and 2.3, or N/PN to 2.4**: Were adjustment techniques used that are likely to correct for the presence of selection biases? |  | N |
|  | **Risk of bias judgement** |  | Moderate |
|  | Optional: What is the predicted direction of bias due to selection of participants into the study? |  | Favours experimental / Favours comparator / Towards null /Away from null / Unpredictable |

| **Bias in classification of interventions** | | | |
| --- | --- | --- | --- |
|  | 3.1 Were intervention groups clearly defined? |  | Y |
|  | 3.2 Was the information used to define intervention groups recorded at the start of the intervention? |  | Y |
|  | 3.3 Could classification of intervention status have been affected by knowledge of the outcome or risk of the outcome? |  | PN |
|  | **Risk of bias judgement** |  | Low |
|  | Optional: What is the predicted direction of bias due to classification of interventions? |  | Favours experimental / Favours comparator / Towards null /Away from null / Unpredictable |

| **Bias due to deviations from intended interventions** | | | |
| --- | --- | --- | --- |
|  | **If your aim for this study is to assess the effect of assignment to intervention, answer questions 4.1 and 4.2** | |  |
|  | 4.1. Were there deviations from the intended intervention beyond what would be expected in usual practice? |  | PN |
|  | 4.2. **If Y/PY to 4.1**: Were these deviations from intended intervention unbalanced between groups *and* likely to have affected the outcome? |  | NA / Y / PY / PN / N / NI |
|  | **If your aim for this study is to assess the effect of starting and adhering to intervention, answer questions 4.3 to 4.6** | |  |
|  | 4.3. Were important co-interventions balanced across intervention groups? |  | Y / PY / PN / N / NI |
|  | 4.4. Was the intervention implemented successfully for most participants? |  | Y / PY / PN / N / NI |
|  | 4.5. Did study participants adhere to the assigned intervention regimen? |  | Y / PY / PN / N / NI |
|  | 4.6. **If N/PN to 4.3, 4.4 or 4.5**: Was an appropriate analysis used to estimate the effect of starting and adhering to the intervention? |  | NA / Y / PY / PN / N / NI |
|  | **Risk of bias judgement** |  | Low |
|  | Optional: What is the predicted direction of bias due to deviations from the intended interventions? |  |  |

| **Bias due to missing data** | | | |
| --- | --- | --- | --- |
|  | 5.1 Were outcome data available for all, or nearly all, participants? |  | Y |
|  | 5.2 Were participants excluded due to missing data on intervention status? |  | N |
|  | 5.3 Were participants excluded due to missing data on other variables needed for the analysis? |  | N |
|  | 5.4 **If PN/N to 5.1, or Y/PY to 5.2 or 5.3**: Are the proportion of participants and reasons for missing data similar across interventions? |  | NA / Y / PY / PN / N / NI |
|  | 5.5 **If PN/N to 5.1, or Y/PY to 5.2 or 5.3**: Is there evidence that results were robust to the presence of missing data? |  | NA / Y / PY / PN / N / NI |
|  | **Risk of bias judgement** |  | Low |
|  | Optional: What is the predicted direction of bias due to missing data? |  | Favours experimental / Favours comparator / Towards null /Away from null / Unpredictable |

| **Bias in measurement of outcomes** | | | |
| --- | --- | --- | --- |
|  | 6.1 Could the outcome measure have been influenced by knowledge of the intervention received? |  | PN |
|  | 6.2 Were outcome assessors aware of the intervention received by study participants? |  | PY |
|  | 6.3 Were the methods of outcome assessment comparable across intervention groups? |  | Y |
|  | 6.4 Were any systematic errors in measurement of the outcome related to intervention received? |  | PN |
|  | **Risk of bias judgement** |  | Low / Moderate / Serious / Critical / NI |
|  | Optional: What is the predicted direction of bias due to measurement of outcomes? |  | Favours experimental / Favours comparator / Towards null /Away from null / Unpredictable |

| **Bias in selection of the reported result** | | | |
| --- | --- | --- | --- |
|  | Is the reported effect estimate likely to be selected, on the basis of the results, from... |  |  |
|  | 7.1. ... multiple outcome *measurements* within the outcome domain? |  | PN |
|  | 7.2 ... multiple *analyses* of the intervention-outcome relationship? |  | N |
|  | 7.3 ... different *subgroups*? |  | N |
|  | **Risk of bias judgement** |  | Low |
|  | Optional: What is the predicted direction of bias due to selection of the reported result? |  | Favours experimental / Favours comparator / Towards null /Away from null / Unpredictable |

| **Overall bias** | | | |
| --- | --- | --- | --- |
|  | **Risk of bias judgement** |  | Low |
|  | Optional: What is the overall predicted direction of bias for this outcome? |  | Favours experimental / Favours comparator / Towards null /Away from null / Unpredictable |

**Risk of bias assessment Krasniqi et al. 2012**

Responses underlined in green are potential markers for low risk of bias, and responses in red are potential markers for a risk of bias. Where questions relate only to sign posts to other questions, no formatting is used.

|  | **Signalling questions** | | **Description** | **Response options** |
| --- | --- | --- | --- | --- |
| **Bias due to confounding** | | | | |
|  | 1.1 Is there potential for confounding of the effect of intervention in this study?  **If N/PN to 1.1:** the study can be considered to be at low risk of bias due to confounding and no further signalling questions need be considered | |  | Y |
|  | **If Y/PY to 1.1**: determine whether there is a need to assess time-varying confounding: | |  |  |
|  | 1.2. Was the analysis based on splitting participants’ follow up time according to intervention received?  **If N/PN**, answer questions relating to baseline confounding (1.4 to 1.6)  **If Y/PY**, go to question 1.3. | |  | N |
|  | 1.3. Were intervention discontinuations or switches likely to be related to factors that are prognostic for the outcome?  **If N/PN**, answer questions relating to baseline confounding (1.4 to 1.6)  **If Y/PY**, answer questions relating to both baseline and time-varying confounding (1.7 and 1.8) | |  | NA / Y / PY / PN / N / NI |
|  | **Questions relating to baseline confounding only** | | | |
|  | 1.4. Did the authors use an appropriate analysis method that controlled for all the important confounding domains? |  | N | |
|  | 1.5. **If Y/PY to 1.4**: Were confounding domains that were controlled for measured validly and reliably by the variables available in this study? |  | NA / Y / PY / PN / N / NI | |
|  | 1.6. Did the authors control for any post-intervention variables that could have been affected by the intervention? |  | N | |
|  | **Questions relating to baseline and time-varying confounding** | |  | |
|  | 1.7. Did the authors use an appropriate analysis method that controlled for all the important confounding domains and for time-varying confounding? |  | N | |
|  | 1.8. **If Y/PY to 1.7**: Were confounding domains that were controlled for measured validly and reliably by the variables available in this study? |  | NA / Y / PY / PN / N / NI | |
|  | **Risk of bias judgement** |  | Serious | |
|  | Optional: What is the predicted direction of bias due to confounding? |  | Favours experimental / Favours comparator / Unpredictable | |

| **Bias in selection of participants into the study** | | | |
| --- | --- | --- | --- |
|  | 2.1. Was selection of participants into the study (or into the analysis) based on participant characteristics observed after the start of intervention?  **If N/PN to 2.1:** go to 2.4 |  | Y |
|  | 2.2. **If Y/PY to 2.1**: Were the post-intervention variables that influenced selection likely to be associated with intervention?  2.3 **If Y/PY to 2.2**: Were the post-intervention variables that influenced selection likely to be influenced by the outcome or a cause of the outcome? |  | PY  PN |
|  | 2.4. Do start of follow-up and start of intervention coincide for most participants? |  | NI |
|  | 2.5. **If Y/PY to 2.2 and 2.3, or N/PN to 2.4**: Were adjustment techniques used that are likely to correct for the presence of selection biases? |  | PN |
|  | **Risk of bias judgement** |  | Serious |
|  | Optional: What is the predicted direction of bias due to selection of participants into the study? |  | Favours experimental / Favours comparator / Towards null /Away from null / Unpredictable |

| **Bias in classification of interventions** | | | |
| --- | --- | --- | --- |
|  | 3.1 Were intervention groups clearly defined? |  | PY |
|  | 3.2 Was the information used to define intervention groups recorded at the start of the intervention? |  | N |
|  | 3.3 Could classification of intervention status have been affected by knowledge of the outcome or risk of the outcome? |  | PN |
|  | **Risk of bias judgement** |  | Moderate |
|  | Optional: What is the predicted direction of bias due to classification of interventions? |  | Favours experimental / Favours comparator / Towards null /Away from null / Unpredictable |

| **Bias due to deviations from intended interventions** | | | |
| --- | --- | --- | --- |
|  | **If your aim for this study is to assess the effect of assignment to intervention, answer questions 4.1 and 4.2** | |  |
|  | 4.1. Were there deviations from the intended intervention beyond what would be expected in usual practice? |  | PN |
|  | 4.2. **If Y/PY to 4.1**: Were these deviations from intended intervention unbalanced between groups *and* likely to have affected the outcome? |  | NA / Y / PY / PN / N / NI |
|  | **If your aim for this study is to assess the effect of starting and adhering to intervention, answer questions 4.3 to 4.6** | |  |
|  | 4.3. Were important co-interventions balanced across intervention groups? |  | Y / PY / PN / N / NI |
|  | 4.4. Was the intervention implemented successfully for most participants? |  | Y / PY / PN / N / NI |
|  | 4.5. Did study participants adhere to the assigned intervention regimen? |  | Y / PY / PN / N / NI |
|  | 4.6. **If N/PN to 4.3, 4.4 or 4.5**: Was an appropriate analysis used to estimate the effect of starting and adhering to the intervention? |  | NA / Y / PY / PN / N / NI |
|  | **Risk of bias judgement** |  | Low |
|  | Optional: What is the predicted direction of bias due to deviations from the intended interventions? |  |  |

| **Bias due to missing data** | | | |
| --- | --- | --- | --- |
|  | 5.1 Were outcome data available for all, or nearly all, participants? |  | N |
|  | 5.2 Were participants excluded due to missing data on intervention status? |  | PN |
|  | 5.3 Were participants excluded due to missing data on other variables needed for the analysis? |  | Y |
|  | 5.4 **If PN/N to 5.1, or Y/PY to 5.2 or 5.3**: Are the proportion of participants and reasons for missing data similar across interventions? |  | NI |
|  | 5.5 **If PN/N to 5.1, or Y/PY to 5.2 or 5.3**: Is there evidence that results were robust to the presence of missing data? |  | NI |
|  | **Risk of bias judgement** |  | Moderate |
|  | Optional: What is the predicted direction of bias due to missing data? |  | Favours experimental / Favours comparator / Towards null /Away from null / Unpredictable |

| **Bias in measurement of outcomes** | | | |
| --- | --- | --- | --- |
|  | 6.1 Could the outcome measure have been influenced by knowledge of the intervention received? |  | PN |
|  | 6.2 Were outcome assessors aware of the intervention received by study participants? |  | PY |
|  | 6.3 Were the methods of outcome assessment comparable across intervention groups? |  | PY |
|  | 6.4 Were any systematic errors in measurement of the outcome related to intervention received? |  | PN |
|  | **Risk of bias judgement** |  | Moderate |
|  | Optional: What is the predicted direction of bias due to measurement of outcomes? |  | Favours experimental / Favours comparator / Towards null /Away from null / Unpredictable |

| **Bias in selection of the reported result** | | | |
| --- | --- | --- | --- |
|  | Is the reported effect estimate likely to be selected, on the basis of the results, from... |  |  |
|  | 7.1. ... multiple outcome *measurements* within the outcome domain? |  | PN |
|  | 7.2 ... multiple *analyses* of the intervention-outcome relationship? |  | PN |
|  | 7.3 ... different *subgroups*? |  | PN |
|  | **Risk of bias judgement** |  | Low |
|  | Optional: What is the predicted direction of bias due to selection of the reported result? |  | Favours experimental / Favours comparator / Towards null /Away from null / Unpredictable |

| **Overall bias** | | | |
| --- | --- | --- | --- |
|  | **Risk of bias judgement** |  | Low / Moderate / Serious / Critical / NI |
|  | Optional: What is the overall predicted direction of bias for this outcome? |  | Favours experimental / Favours comparator / Towards null /Away from null / Unpredictable |
